# A Pocket Laboratory for Functional Neuroimaging Research Using Mobile Visual Oddball, Multimodal Electroencephalography, and Functional Near-Infrared Spectroscopy Imaging: Instrument Validation Study

**DOI:** 10.2196/78217

**Published:** 2026-02-06

**Authors:** Peter Rokowski, Meltem Izzetoglu, Luis Gomero, Roee Holtzer

**Affiliations:** 1 Department of Electrical and Computer Engineering, Villanova University, 800 E. Lancaster Ave, Villanova, PA, 19085, United States, 1 610-519-4500; 2 Ferkauf Graduate School of Psychology, Yeshiva University, New York, NY, United States; 3 Department of Neurology, Albert Einstein College of Medicine, New York, NY, United States

**Keywords:** pocket laboratory, neuroimaging, functional near-infrared spectroscopy, fNIRS, smartphone data collection, mobile brain and body imaging

## Abstract

**Background:**

The need to observe brain activity in more natural environments, that is, outside of laboratory settings, is critical for understanding cognition. Wearable low-cost neuroimaging modalities (electroencephalography [EEG] and functional near-infrared spectroscopy [fNIRS]) are portable, noninvasive, and robust to motion artifacts but lack similarly portable tools for use in ecologically valid studies. Smartphones are ubiquitous, programmable, wireless, and thus strong candidates for “pocket laboratories” companion platforms that travel with study subjects. Therefore, we developed the Wearable Cognitive Assessment and Augmentation Toolkit (WearCAAT), a cross-platform neuroimaging task platform that integrates external sensors via the lab streaming layer (LSL) and supports over 100 sensor types. We validated our implementation with healthy human participants under multimodal neuroimaging conditions prior to analysis of data collection in ongoing clinical settings.

**Objective:**

This study aimed to validate WearCAAT, as a platform for functional neuroimaging research, via analysis of human participant data collected during our ongoing National Institutes of Health–funded study.

**Methods:**

We analyzed data from healthy college-aged (ages 18‐30 y) adult participants, who completed a battery of shortened neurocognitive tasks (each lasting 4 min) in WearCAAT, while outfitted with research-grade multimodal EEG and fNIRS sensors. We indicated validity via the presence of task-related behavioral responses and their neuroimaging correlates. As a representative example, we analyzed the visual oddball task due to its well-documented poststimulus features for EEG and fNIRS. We extracted behavioral responses, mean response accuracies, and response times for infrequent (target) and frequent (standard) stimuli classes. We examined, poststimulus, P300, positive amplitude deflection around 300 (ms) in EEG and increased average oxygenated hemoglobin (HbO) levels in fNIRS.

**Results:**

We enrolled a total of 57 (male individuals: n=27, 47%; female individuals: n=30, 53%; mean age 22, SD 3.4 y) participants for data collection. We excluded the first 4 (7%) participants from our analysis due to technical errors. Our analysis revealed increased mean response times for infrequent (target) stimuli (mean 718, SD 148 ms) compared to frequent (standard) stimuli (mean 542, SD 122 ms) with the Wilcoxon test (Z=6.33; *P*<.001; *r*=0.87); higher P300 amplitudes over midline regions (parietal and temporal) for EEG; and increased oxygenated hemoglobin over the prefrontal cortex for fNIRS. All participants completed the full battery and reported no usability concerns or app crashes. Similarly, we observed no data loss or corruption that would negatively impact analyses.

**Conclusions:**

WearCAAT-provided outcomes from our study, which analyzed multimodal neuroimaging data collected during a mobile app–based visual oddball task, matched expectations from the literature. While full validation is ongoing for other tasks, we demonstrated initial validity of our app for neurocognitive imaging use. Our app and approach represent the first attempt at dedicated neuroimaging mobile-pocket laboratory and contribute to greater studies in ecological validity.

## Introduction

Functional neuroimaging entails the use of noninvasive brain monitoring sensors during tailored neurocognitive tasks to measure cortical activity in different brain regions corresponding to targeted cognitive domains [1]. Neuroimaging research offers views into the brain and is critical to the understanding of brain development, injury, and disease or impairment. High-precision techniques such as functional magnetic resonance imaging have limitations that include participant refusal, high cost, and requirements on staying still in the supine position [2]. This presents an interesting challenge that cognitive neuroscientists have grappled with since the early days of cognitive studies on “ecological validity” [3], wherein experimental conditions, particularly those imposed by the nature of laboratory settings and high-precision imaging modalities such as functional magnetic resonance imaging, impact the observed phenomena [4]. A complementary solution to the challenge of ecological validity is to use wearable, low-power, wireless technologies to combat such challenges facing traditional approaches [5]. Noninvasive wearable neuroimaging modalities include functional near-infrared spectroscopy (fNIRS) to monitor changes in cerebral hemodynamics related to cognitive activity and electroencephalography [EEG] to monitor neural activations via changes in electrophysiology. Both methods enable observations of human participants in more “natural” environments, and their complementary nature allows overcoming of their individual limitations on temporal and spatial resolution when used together [6].

Development and advances in commercially available mobile devices, such as tablets and smartphones, enable portable platforms for conducting human experiments that can further mobilize wearable sensing modalities. The resulting concept “pocket laboratories” describes this paradigm well and poses a unique solution to ecological validity by enabling human participant research in nonstatic environments that could potentially travel with a participant. Researchers in human behavior and medicine have leveraged the widespread adoption of mobile devices as pocket laboratories for more “natural” environments, which are classified under mobile health apps. For example, smartphones and tablets were used in conjunction with neuroimaging devices to measure interactions with websites, apps, and each other, to glean insight into health behaviors such as alcohol consumption [7] and Alzheimer disease detection [8,9]. In addition to the conveniences and benefits that mobile devices offer, they also provide useful hardware for human participant research such as internet connectivity and integrated sensors (ie, gyroscopes, accelerometers, and GPS) [10,11] and are extensible through Bluetooth connections with external wearable health sensors such as the ones embedded in smartwatches. With an estimated 4.7 billion smartphone users by 2024 [12], the potential for participant recruitment is vast, which can further improve not only our understanding of cognition but also allow for the early detection of different conditions, that is, cognitive impairment and monitoring of treatment outcomes through larger studies and populations otherwise unattainable.

Despite the practical uses of mobile devices in clinical research, there’s a notable gap: a platform for conducting general functional neuroimaging research using mobile devices. While accepted tools such as the NIH Toolbox [13] developed by the National Institutes of Health provide a platform for gathering psychometrics on human participants and are widely used in clinical settings (225 journals and conferences as of 2022 [14]), they lack compatibility with functional neuroimaging sensing modalities. Other apps are too limited in scope and lack integration beyond basic proof of concepts and require significant effort to extend to new scenarios. We believe this is because app development is difficult and requires deep technical knowledge and funding that is outside the scope of normal external funding vehicles [15]. In response to this, we developed a framework for a functional neuroimaging pocket laboratory and provided implementation in the Wearable Cognitive Assessment and Augmentation Toolkit (WearCAAT).

WearCAAT is a cross-platform mobile app, used on both iOS and Android, in conjunction with external single or multimodal sensors, integrated via the lab streaming layer (LSL) [16]. LSL adds signal synchronization capabilities, equivalent on mobile devices to desktop systems [17]. However, validation of our framework and implementation is still outstanding. There are numerous challenges in translating a desktop software capability to mobile devices, especially in the domain of functional neuroimaging. Touchscreens are dual-purpose tools that share the responsibility of presenting stimuli and capturing responses via “soft” buttons. Mobile operating systems are sandboxed in nature and typically prevent access to high-precision time-aware clocks, as well as limit multithreading capabilities. A full end-to-end test for our paradigm is necessary to understand the limits and abilities of pocket laboratories in functional neuroimaging.

## Methods

### Ethical Considerations

Participants signed informed consent before completing cognitive tasks using WearCAAT on an iPad. The consent and collection protocol were reviewed and approved by the Biomedical Research Alliance of New York, LLC (BRANY) [18], external institutional review board (1R01AG077018-01), on March 24, 20. Participant privacy was covered under the certificate of confidentiality by the National Institutes of Health that states that researchers will not disclose or use information that may identify participants in any federal, state, or local civil, criminal, administrative, legislative, or other action, suit, or proceeding, even if there is a court subpoena (with exceptions being federal, state, or local law that requires disclosure, or the explicit approval of individual participants to release their name and/or personally identifiable information). Participants were compensated US $20.

### Procedure

We examined whether neurocognitive tasks provided by WearCAAT, on commercial mobile devices, reliably elicited cognitive engagement and whether the corresponding biological markers were detectable and identifiable in neuroimaging data. This required that (1) behavioral responses aligned with established task-specific patterns in the literature, and (2) these responses enabled the extraction of physiologically meaningful signals from neuroimaging modalities. Failure to meet both criteria across tasks constituted a negative inconclusive finding, whereas partial success supported the technical validity of our implementation and integration. Consequently, we scoped our analysis on the visual oddball paradigm [19], also built in WearCAAT, which is well studied in both EEG and fNIRS modalities for attention monitoring with established expected behavioral, neural, and hemodynamic outcomes [20-23].

First, we hypothesized that participants’ behavioral data during the visual oddball task, as built in WearCAAT, would exhibit a longer response time (RT) to infrequent (target) stimuli than to frequent (standard) stimuli. Second, we hypothesized that neural correlates for cognition, as measured by EEG and fNIRS during the performance of the visual oddball task, would be detectable in their respective sensing modality when examined using time stamps obtained from the behavioral data. For EEG, the WearCAAT-implemented visual oddball task would evoke a higher P300 subcomponent (positive deflection in amplitude around 300 ms after stimulus) in event-related potentials (ERPs; stimulus-locked activations in EEG) to the infrequent (target) stimuli as compared to the frequent (standard) ones in the midline region. For fNIRS, the average oxygenated hemoglobin (HbO) would positively increase in response to infrequent (target) stimuli as opposed to the frequent (standard) stimuli, in the right prefrontal cortex (PFC).

The remainder of this section describes WearCAAT and the relevant features of this study, followed by the participant information, data collection protocol, the visual oddball task as presented in WearCAAT, and the signal processing pipeline to extract the EEG- and fNIRS-specific components that support or reject our hypotheses.

### WearCAAT: An Overview

WearCAAT implements task-based neurocognitive monitoring, wherein participants perform 1 of 11 built-in tasks to elicit known cognitive effects in different domains, including attention, vigilance, working and episodic memory, response inhibition, set shifting, and conflict resolution. Currently implemented tasks in WearCAAT and their cognitive effects are presented in Table 1. We built WearCAAT using C# and Extensible Application Markup Language (XAML) from the Multi-App User-Interface (MAUI) framework [24] with .NET 8. MAUI provides cross-platform (supporting Android and iOS phones and tablets) app design in a unified project. LSL is integrated using the “slim bindings” approach for high-performance C and C++ libraries to be leveraged through different programming languages, giving direct access to the necessary libraries.

**Table 1.** Currently existing cognitive task battery implemented in the Wearable Cognitive Assessment and Augmentation Toolkit. Cognitive battery has a total runtime of approximately 1.5 hours, and each task had a runtime of 4 minutes with a 30-second rest period in between, which can be implemented in a randomized order.

Task name	Cognitive effect
Psychomotor vigilance task [25,26]	Attention or vigilance
Visual oddball paradigm [19]	Attention or working memory
Go/no-go [27]	Response inhibition
N-back (n = [0, 1, 2]) [28,29]	Working memory
Stroop [30]	Selective attention
Flanker [31,32]	Conflict resolution
Wisconsin card sorting [33]	Set shifting
Verbal memory recognition [34]	Episodic memory
Bluegrass [35]	Episodic memory
Resting (eyes = [open, closed]) [36]	Default mode net

**Figure 1.**
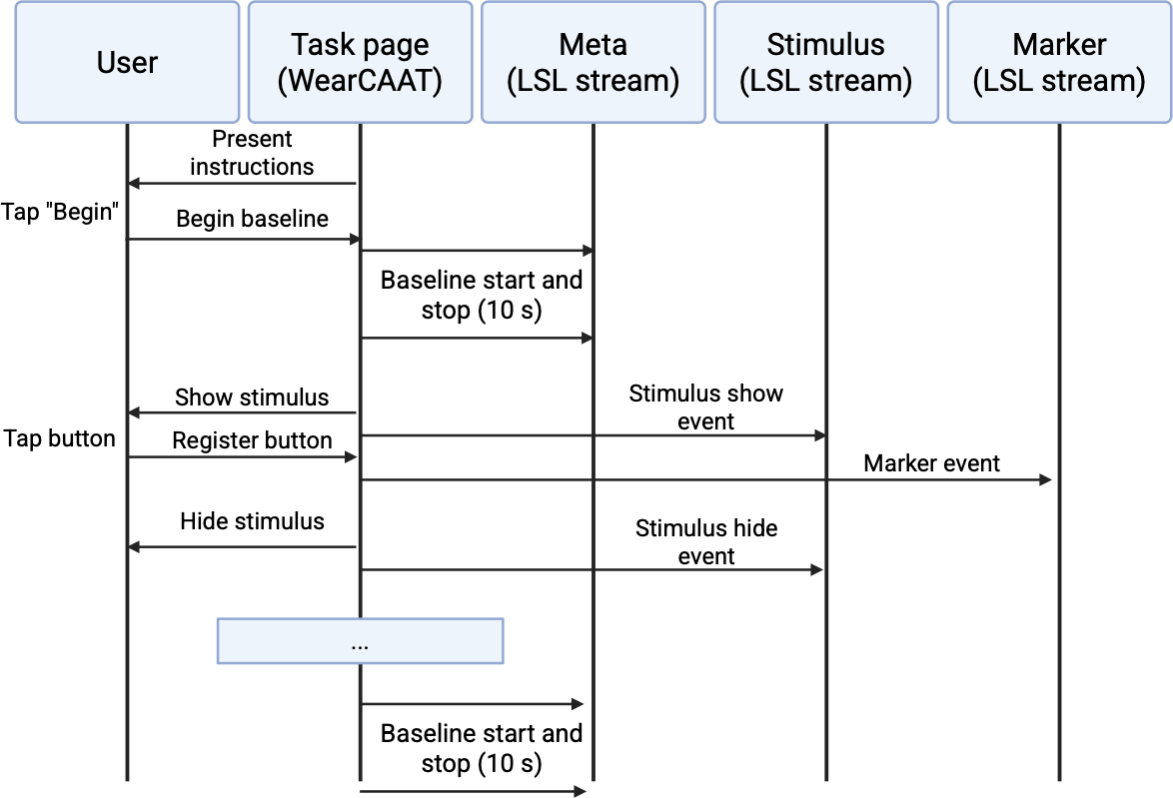
Task sequence diagram. Baseline periods begin immediately, with all events related to the task going through to LSL streams. “Task page” represents the logic controller behind the task in WearCAAT (created in BioRender [37]). LSL: lab streaming layer; WearCAAT: Wearable Cognitive Assessment and Augmentation Toolkit.

Neurocognitive tasks followed the sequence described in Figure 1. The participant first read the instructions, then started the assessment via tapping the “begin” button, upon which the screen was blank for a configurable resting baseline time window before the task’s logic loop began. The task expired after the set amount of time and was followed by a second baseline period. Timing information was determined using a stopwatch object, which counted monotonically from the start, as is common in psychometrics platforms [16]. Tasks were configurable in the app to allow further flexibility and experimentation, specifically regarding stimulus type, timing, interstimulus interval information, stimulus presentation ratios, and more, depending on the task.

Task events were broken down into three categories as follows: (1) metadata pertaining to task information and configuration for auditing purposes; (2) stimulus event appearances, types, etc; and (3) user markers, button presses, or other responses. Each category streamed data to a corresponding LSL stream outlet, which sent the data wirelessly to the recording platform. Figure 1 depicts the sequence of events and the respective streams for each task.

### Participants

We recruited 57 (male individuals: n=27, 47%; female individuals: n=30, 53%) participants, aged 18 to 30 (mean age 22, SD 3.4 y) years, from the undergraduate and graduate student bodies at Villanova University via flyers posted in common university spaces. We detail participant demographic data in Table 2. Exclusion criteria included current or past severe neurological or psychiatric disorders and significant vision or hearing impairments. Participants first attended an initial screening, where we collected demographic data and relevant medical histories via a written survey, measured the participant’s head circumference to determine neuroimaging device cap size, and scheduled a follow-up data collection session. After participants signed the informed consent form, we collected behavioral and neuroimaging data from them while they used WearCAAT in a session that took approximately 1.5 hours.

**Table 2.** Demographic data of recruited college-aged participants.

Demographics	Male (n=27), n (%)	Female (n=30), n (%)	Total (N=57), n (%)
Asian	8 (30)	7 (23)	15 (26)
Black or African American	1 (4)	0 (0)	1 (2)
Hispanic	2 (7)	5 (17)	7 (12)
White	13 (48)	13 (43)	26 (46)
White and Black or African American	2 (7)	0 (0)	2 (4)
White and Hispanic	1 (4)	5 (17)	6 (11)

### Data Collection

**Figure 2.**
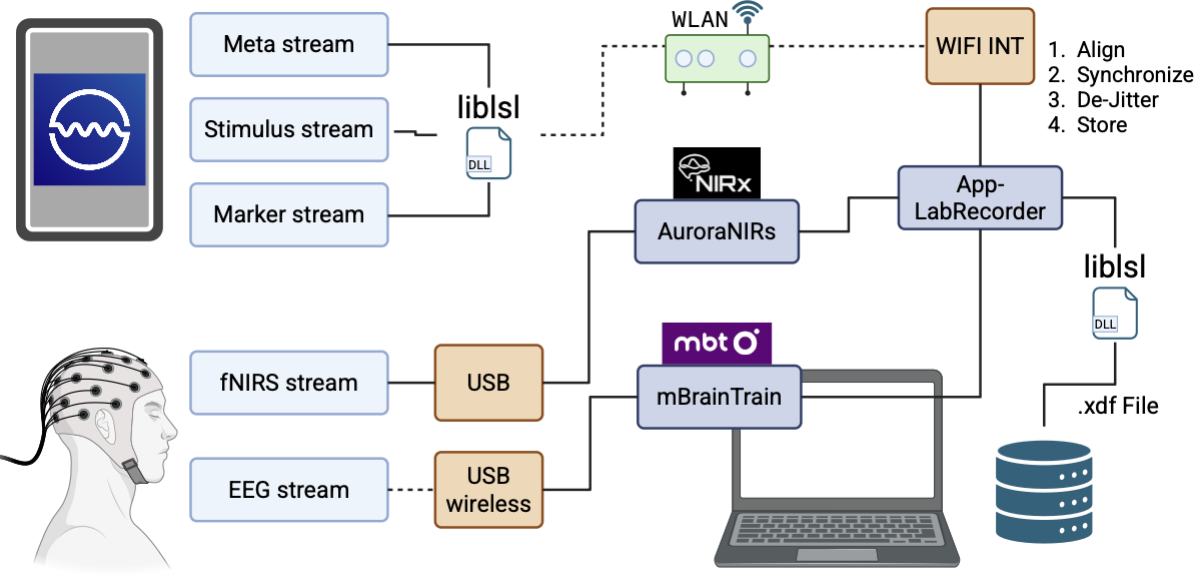
Lab streaming layer event pipelines in the Wearable Cognitive Assessment and Augmentation Toolkit; buttons, stimuli, and metadata streamed wirelessly using the embedded liblsl library to the laptop via an onboard wireless interface; fNIRS connected via wired USB-cable collected using AuroraNIRS; EEG streamed via dedicated wireless dongle; data are saved to XDF files. DLL: Dynamic-Link Library; EEG: electroencephalogram; fNIRS: functional near-infrared spectroscopy; WLAN: wireless-local area network; XDF: extensible data format.

We administered all 11 abbreviated tasks to participants via WearCAAT in sequence, where the first 2 were the resting tasks (eyes opened and eyes closed) and the remaining 9 tasks were presented in randomized order (Table 3). All participants completed the entire task battery built in WearCAAT in 1 sitting while wearing a full head cap housing the multiple optodes. The electrodes formed a combined wireless fNIRS-EEG system (NIRSport2, NIRx Medizintechnik GmbH and Smarting-mbt wireless EEG, mBrainTrain, respectively) [38,39]. Using our hybrid neuroimaging system, we collected 51-channel fNIRS (43 long and 8 short distances) and 32-channel EEG data from the frontal, temporal, and parietal regions of the brain simultaneously. We used 3 distinct flexible cap sizes as provided by NIRx company [40]—small (56 cm), medium (58 cm), and large (60 cm)—all with 128 slits for probe locations identified according to the 10-20 international system to accommodate for different head sizes, improve comfort, and ensure fNIRS and EEG measurements with good coupling from similar head locations. The complete layout of our protocol is detailed in Figure 2; the LSL data streams and their respective interfaces are all coordinated through a Wireless Area Network (WAN) hosted on a private router with no external internet connection nor devices.

**Table 3.** The full mapping of electroencephalography (EEG) and functional near-infrared spectroscopy (fNIRS) sensor locations to functional regions of interest (ROI). For our analysis, we considered regions over the prefrontal cortex, specifically the “frontal right” ROI for fNIRS. The “frontal” (Fz), “parietal” (Pz), and “temporal” (Cz) midlines are areas we focus on to observe the P300 for EEG.

ROI	EEG channels	fNIRS channels
Frontal left	AFP1 AFF5 F3 F1	FPZ-FP1, FPZ-AF3 FF7-AF3 F5-AF3, F5-F7 AF3-AFz
Frontal right	AFP2 AFF6h F4 F2	FPZ-FP2, FPZ-AF4 AF8-FP2, AF8-AF4 F6-AF4, F6-F8 AF4-AFz
Temporal left	FTT7h TTP7h	FT8-T8 TP8-T8 C6-T8
Temporal right	FTT8h TTP8h	FT7-T7 TP7-T7 C5-T7
Parietal left	P1, P7 CPP5h TPP8h	P5-P3, P5-C5 P3 CP3
Parietal right	P2, P8 CPP6h TPP8h	P6-P4, P6-CP6 P4 CP4
Frontal midline	Fz	—^a^
Parietal midline	Pz	—
Temporal midline	Cz	—

a Not available.

Behavioral responses were recorded in WearCAAT where task events (eg, stimulus presentation times, user responses via button presses, etc) were time stamped in WearCAAT and wirelessly streamed through LSL to a laptop (Windows 10) running App-LabRecorder [41]. Concurrent EEG and fNIRS data were also streamed wirelessly to the same laptop, which synchronized all clock times and removed jitter automatically. While WearCAAT supports Android, we only used an Apple iPad Pro (sixth generation) as our mobile platform due to the consistency of iOS devices. Using different operating systems and devices from different manufacturers might introduce errors that are harder to quantify [42], and that is out of scope for this body of work.

### Evaluation Protocol: Visual Oddball Paradigm

**Figure 3.**
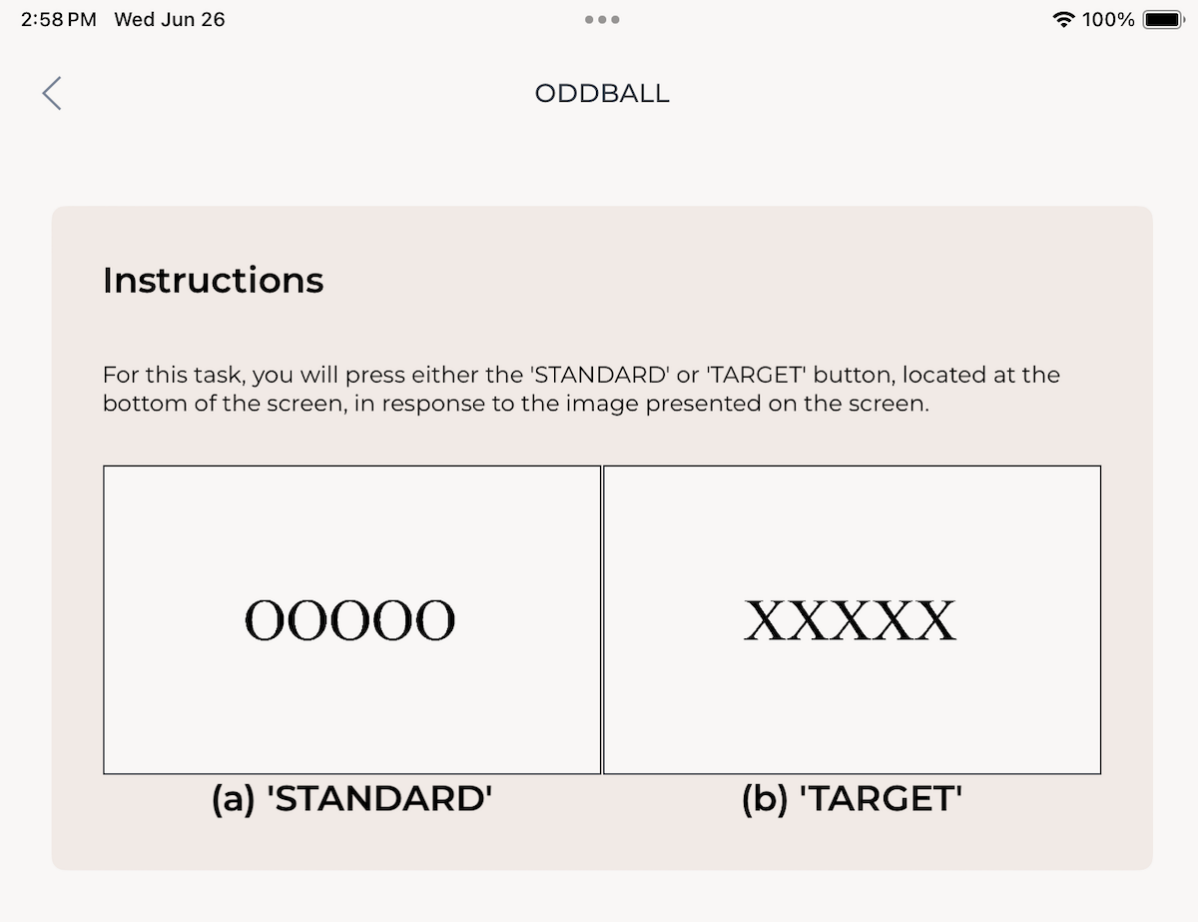
In-app instructions for the visual oddball task and examples of stimuli. (****A****) STANDARD stimulus on the left (OOOOO) and (**B**) TARGET stimulus on the right (XXXXX). Stimuli are presented as rectangles with black font on a white background.

To provide empirical support and initial validation, we reported our neuroimaging and behavioral outcomes obtained from the abbreviated visual oddball task implemented via WearCAAT. In this task, participants were presented with either 1 of 2 types of visual stimuli where each consisted of 5 repeated letters and asked to respond by tapping 1 of the 2, parallel and equally sized, buttons at the bottom of the screen with the index finger on their dominant hand. The target stimulus (“XXXXX”) matched with the left-most button labeled “TARGET,” and the standard stimulus (“OOOOO”) matched to the right-most button. The interstimulus interval was 2 seconds, with stimuli presentations lasting 0.5 seconds and the screen remaining blank for 1.5 seconds. Target stimuli appeared infrequently relative to the standard, with at least 7 to 21 standard stimuli appearing between each target presentation to reduce participant expectation. On average, each participant witnessed 11.87 (SD 1.15) standard stimuli between each successive target stimulus. The total duration of the task was 4 minutes and occurred between two 10-second baseline periods. Participants received instructions verbally from the experimenters and in the app before beginning each task, as depicted in Figure 3. Participants began the task by tapping the “begin” button, which started the baseline period followed by the oddball sequence.

### Signal Processing

#### Overview

**Figure 4.**
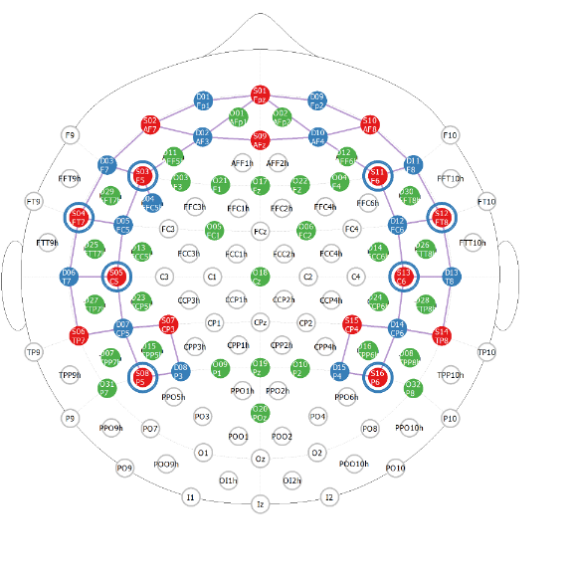
Our hybrid cap design based on the modified 10-20 layout provided by NIRx. Electroencephalogram sensors are depicted in green, functional near-infrared spectroscopy (fNIRS) source in blue, and fNIRS detectors in red. fNIRS short channel locations are shown with blue circles around the red sources.

The simultaneous 51-channel fNIRS and 32-channel EEG data were collected from the full head in frontal, temporal, and parietal locations as shown in Figure 4, with regions of interest (ROI) detailed in Table 3. Our fNIRS and EEG data processing pipeline for artifact removal (motion, physiological, environmental, or equipment noise) and data conversion (hemodynamic response extraction) was performed offline using custom-built MATLAB (version R2024; MathWorks, Inc) codes [43] and in accordance with published best practices [44-46]. We used the 8 fNIRS short channels to remove skin artifacts. EEG data were additionally processed for the removal of eye blinks, eye movement, muscle artifacts, power line noise, and limiting the data within the range of 0 Hz to 45 Hz.

Given the large number of channels (32 for EEG and 43 long separations for fNIRS), we organized the data into ROIs based on both anatomical and functional considerations (detailed in Table 3). To manage the data efficiently, we averaged the responses for each stimulus (target and standard) separately across all 57 participants, focusing on “valid” data from each participant, channel, and stimulus. We determined a channel to be invalid based on impedance for EEG (>30 kΩ) [47], and Scalp Coupling Index (<0.4) for fNIRS [48]. Finally, we averaged the score per channel across all participants and grouped the channels based on their respective ROIs, reducing the dimensionality of the dataset.

#### WearCAAT Markers: User Behavioral Responses

We gathered the response accuracy by evaluating the correct and incorrect user responses and extracting the reaction times to target and standard stimuli. We determined significance between target and standard responses using the Wilcoxon test, a nonparametric alternative to the paired 2-tailed *t* test when the data do not follow a normal distribution.

#### EEG: Event-Related Potential

We extracted stimulus-locked data epochs (ERPs) from EEG recordings using 0.2-second prestimulus and 1-second poststimulus interval and performed a baseline correction by subtracting the mean of the prestimulus data from the whole data epoch. We then removed intrinsic response variability by averaging multiple trials of epochs within the task to obtain an averaged ERP for each stimulus (target and standard), separately. Finally, we gathered the ERP features by extracting the positive and negative peak amplitudes with their respective timings, specifically focusing on the P300 component (positive deflection around 300 ms after the stimulus), which was shown to be higher in target response as compared to the standard one in the visual oddball task in healthy young adults [49,50].

#### fNIRS: Oxygenated Hemoglobin

We processed our fNIRS data by removing motion artifacts and baseline shifts through wavelet and spline filters [44]. We further removed physiological signals, cardiac, respiratory, and Mayer waves, with a finite impulse response low-pass filter using the cutoff frequency 0.08 Hz [51]. Finally, we converted the light intensity measurements into changes in HbO and deoxygenated hemoglobin using the modified Beer-Lambert Law with published coefficients from the literature [52]. After using short channel recordings to remove potential skin blood flow artifacts from long channel recordings using a general linear model [53], we extracted 20-second poststimulus data epochs and applied baseline correction using the 1-second prestimuli onset. Notably, since HbO was the most used fNIRS measure in studies implementing the visual oddball task that was indicative of cognitive activity–related changes in attention domain–specific apps [23], we focused our results and comparisons to only HbO outcomes in this study.

## Results

### Overview

**Figure 5.**
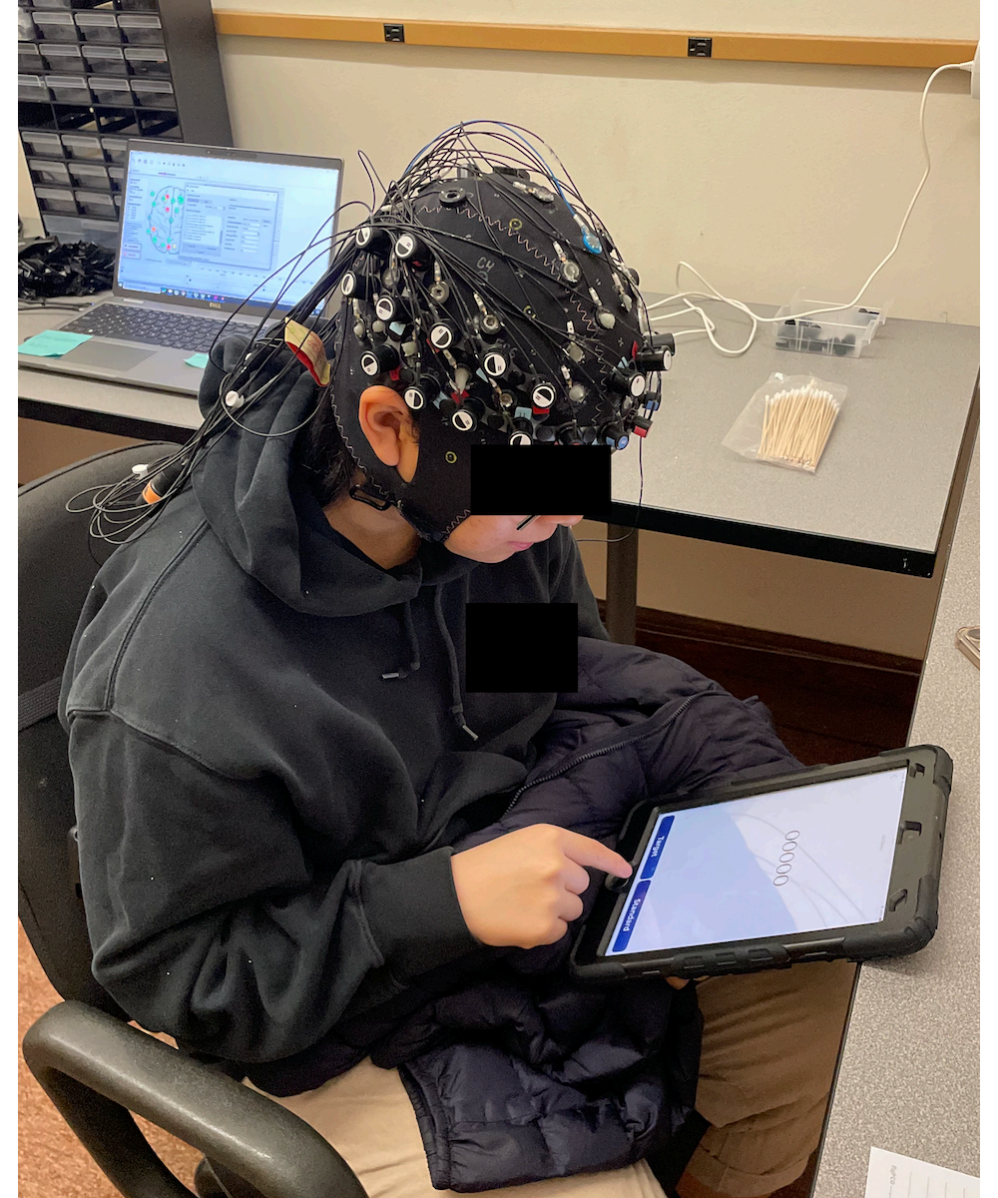
Participant during data collection, responding to a standard (infrequent) stimulus presented via the Wearable Cognitive Assessment and Augmentation Toolkit.

We enrolled 57 participants in data collection, and all of them performed all 11 tasks in 1 sitting. We observed zero participant dropout with no app crashes or corrupted data. We excluded data from our first 4 (7%) participants; 2 due to poor impedance from improper cap setup, and 2 after a patch to WearCAAT that altered the timing logic to improve the responsiveness of the touch screen during timed loops. WearCAAT collected and synchronized multiple concurrent streams of task-related data (1 stream for stimulus; 1 for each button press; and 1 for metadata and task events, such as task start and stop and baseline start and stop) with no data loss in participant responses or disconnects from the recording server. We also observed no additional loss of information or signal content from the combined fNIRS-EEG sensors as well. Participants reported no complaints or concerns with the WearCAAT app, the instructions provided either in app or verbally, or the overall data collection procedure, indicating a low participant burden. An example participant can be seen sitting comfortably during collection in Figure 5.

### User Response Times

We found that the mean RT for target stimuli was 718 (SD 148) milliseconds and, for standard stimuli, it was 542 (SD 122) milliseconds. The Wilcoxon test revealed significant differences in RT to target (infrequent) stimuli as compared to the standard (frequent) ones (Z=6.33; *P*<.001; *r*=0.87). These outcomes indicated that the participants took longer to identify the target stimulus than the standard stimulus. Participants’ mean percent accuracy for identifying stimuli was 94.58 (SD 7.412) for the target stimulus and 99.12 (SD 1.447) for the standard stimulus, suggesting that participants identified and responded to the frequent stimulus more correctly as compared to the infrequent ones overall.

### EEG: Extracted P300

**Figure 6.**
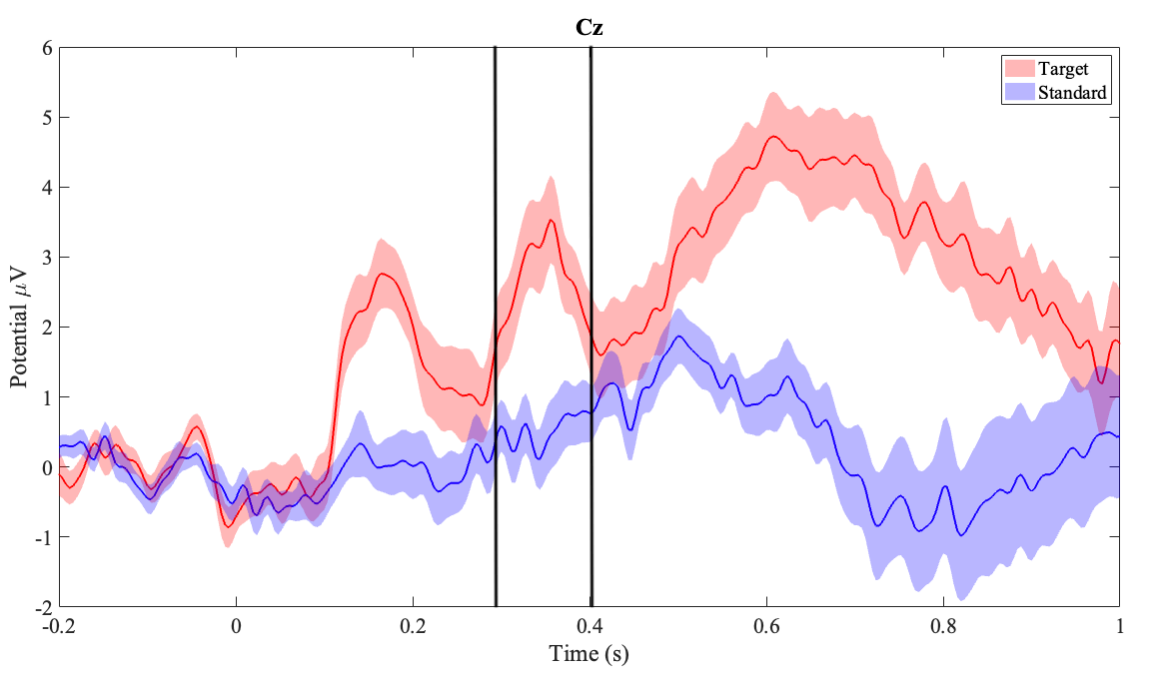
Mean amplitudes across participants in the central midline (Cz). Clouded regions represent the standard error of the mean. Red represents target (infrequent) stimulus and blue represents (frequent) stimulus responses.

All participant-averaged (SEM) ERP waveforms obtained using WearCAAT in an iPad for the abbreviated visual oddball task for target (red) and standard (blue) stimulus in the parietal midline (Pz) and central midline (Cz) regions are presented in Figure 6, separately. Our results showed higher P300 amplitude in response to target stimuli as compared to the standard one, especially in the midline regions on the Cz and Pz locations, in line with the published literature on computerized presentation of a regular length (approximately 20 min) visual oddball task. The Cz, presented in Figure 6, recorded a positive peak within the 250-millisecond to 400-millisecond intervals at 356 milliseconds, having an amplitude of 3.5397 (SD 0.6258) μV for the target stimulus and at 372 milliseconds with an amplitude of 0.74667 (SD 0.4929) μV for the standard stimulus.

**Figure 7.**
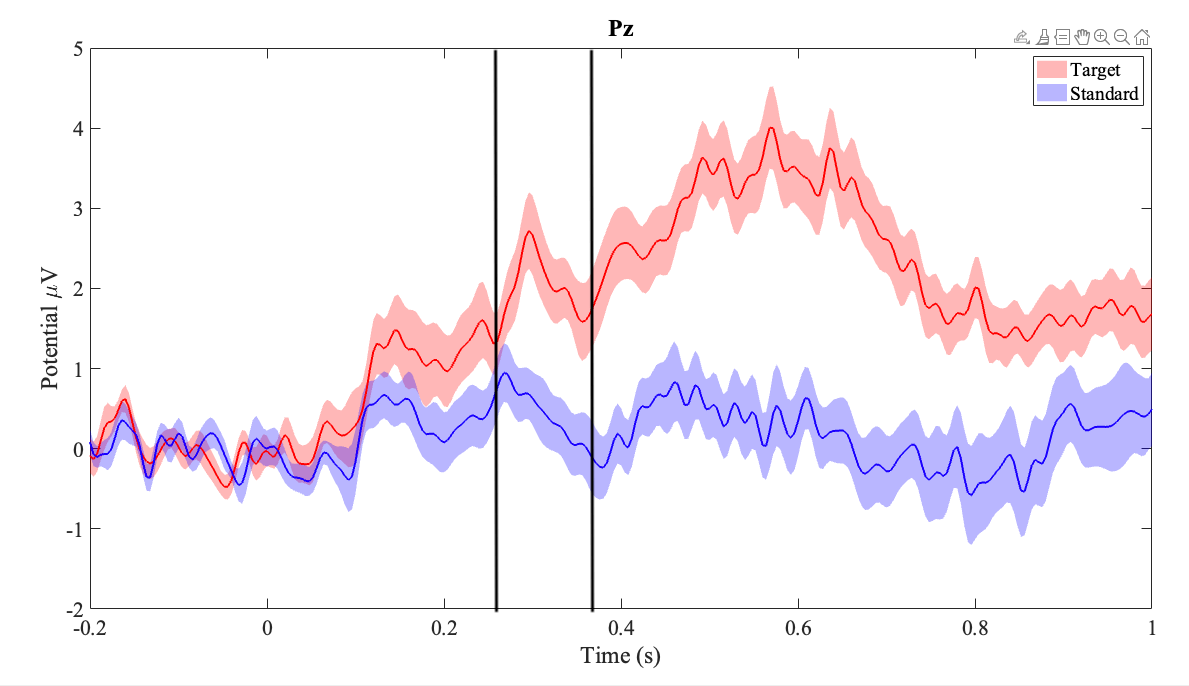
Mean amplitudes across participants in the parietal midline (Pz). Clouded regions represent the standard error of the mean. Red represents target (infrequent) stimulus and blue represents (frequent) stimulus responses.

Similarly, the Pz, presented in Figure 7, recorded a peak at 296 ms with an amplitude of 2.7219 (SD 0.4756) μV in the target stimulus and a peak at 268 ms with an amplitude of 0.949 (SD 0.3637) μV in the standard stimulus.

### fNIRS: Trends in Oxygenated Hemoglobin

**Figure 8.**
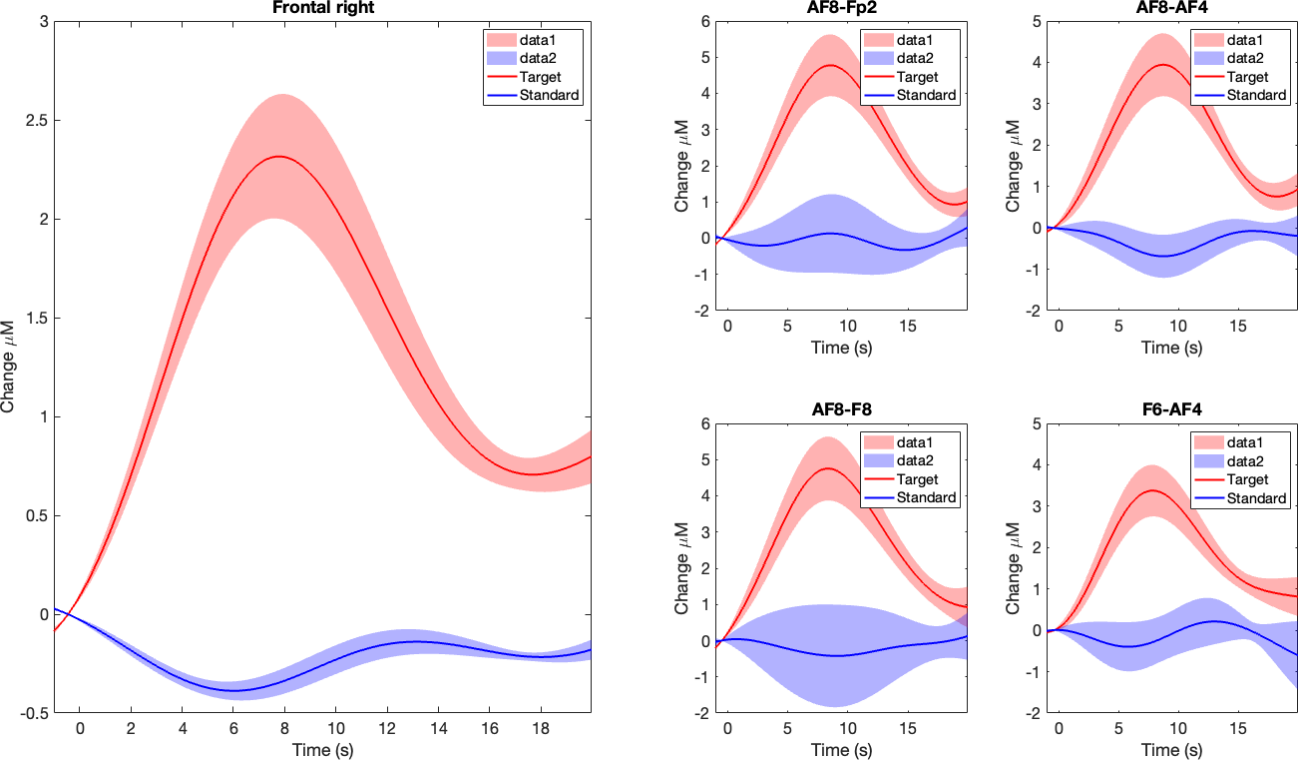
Mean oxygenated hemoglobin activations in the frontal right region of interest across all participants. Clouded regions represent the standard error of the mean. Red represents the target (infrequent) stimulus and blue represents the (frequent) stimulus responses. The aggregate frontal right forehead region is displayed on the left, and the individual sensors of interest are displayed in the quadrant on the right.

As presented in Figure 8, the overall participant-averaged (SEM) HbO activations in the right PFC demonstrated a clear, positive increase in response to the target (red) stimulus peaking at approximately 9 seconds and then returning to lower values following the shape of a typical hemodynamic response function. The standard stimulus (blue) did not generate an increased HbO pattern.

Same trends on HbO in response to target and standard stimuli were observed in all right frontal fNIRS channels constituting the right frontal ROI, as also presented separately in Figure 8.

## Discussion

### Principal Findings

We presented our analysis for the abbreviated visual oddball task as presented and collected using WearCAAT and our procedures. We found indications that electrophysiological and hemodynamic activation patterns for the brain observed with simultaneously collected fNIRS and EEG data follow expected trends, despite the shorter runtime (4 min as opposed to 20 min in commonly implemented versions of the task) and the mobile app platform (as opposed to a computer screen).

Our initial hypothesis for the behavioral responses was supported. We observed greater RTs to target (infrequent) versus standard (frequent) stimuli. The Wilcoxon signed-rank test demonstrated the significance for RT between infrequent and frequent stimuli. These findings are consistent with previously reported values in the literature for the visual oddball task [54,55].

Interestingly, we also observed that periods of responses for target stimuli were greater than some reported values, whereas the standard stimuli RTs were much closer. This may be attributed, in part, to the physical differences in iPad “soft” buttons and the typical hardware switches commonly used with the visual oddball task [20]. Traditional desktop setups report participants using 2 distinct controllers (1 per hand), which dedicate a controller response per stimulus type. In contrast with our study, participants used a singular index finger to switch between button presses. The typical delay reported between stimulus types could be exacerbated by physical delays introduced by a participant needing to move his finger from hovering over 1 button to another one on the opposite side of the iPad screen. As future work, we will ensure that participants are instructed on how to hold the iPad, with relevant findings from the literature.

Our second hypothesis regarding EEG was supported by the successful extraction of P300 subcomponents from the ERP waveform, described as a positive deflection in amplitude in response to the target stimulus, appearing around 300 milliseconds after the stimulus from our collected data. We obtained higher P300 amplitude in the midline ROIs (Pz and Cz) for the infrequent (target) stimuli as compared to the frequent (standard) stimuli, following the expected outcome as reported in the literature [20,21,54,56]. Our average latencies for P300 peaks in Pz and Cz were also within previously reported bounds [57].

We noted the visual jaggedness of the P300 signals, seen in Figures 6 and 7, which we expected to be smoother, as reported in the literature. This could be caused by a combination of the shortened task times and the grand averaging technique used for analysis. Typical studies report task lengths of 20 minutes or greater for the visual oddball task, whereas this study’s task length was 4 minutes. Longer task times would produce 5 times more trials for both target and standard stimuli per participant, the averages of which would smooth out irregularities and potential physiological artifacts in the time series. Further study using WearCAAT, EEG, and the visual oddball task with longer task times would provide more clarity on the matter.

Our second hypothesis regarding fNIRS was also supported by the positive average HbO increases measured in the right PFC in response to infrequent (target) stimuli as compared to the frequent (standard) stimuli. Specifically, we observed the increases in HbO in the frontal right ROI, which was widely reported in the fNIRS literature where computerized and traditional length visual oddball task was used [20,23]. In fact, such findings were prominent in all right frontal channels when considered separately as well as demonstrating an attention domain–specific global activation in right PFC as measured by fNIRS.

On usability, we point to the smoothness of data collection. Specifically, the lack of complaints from the participants and experimenters, combined with the zero-dropout rate and app crashes, is to be noted. Given that users’ major concern with mobile health apps is the perceived bugginess and clunkiness of apps [15], we incorporated haptics and button color changes as feedback to users’ actions. We assume the responsiveness and perceived functionality of our WearCAAT implementation is tolerable to young adults who are most fluent and comfortable in app use. However, because we did not perform a formal qualitative post–data collection survey, our interpretation is limited to “no complaints were reported.” This limitation ought to be accounted for in future studies with formal participant surveys after participation to add qualitative metrics for perceived clunkiness and usability.

### Conclusions

In this study, we provided evidence for the technical validation of mobile devices in task-based functional neuroimaging research via the analysis of multimodal EEG-fNIRS and behavioral data collected during an abbreviated mobile visual oddball task from 57 healthy young adults. Specifically, our goal was to evaluate whether behavioral effects, higher mean responses to infrequent (target) versus frequent (standard) stimuli, were present across participants. We also determined if the P300 component obtained from the ERP waveform on the midline and increases in measured HbO over the right PFC, as measured by fNIRS for target stimuli as compared to the standard ones, can be simultaneously captured using the visual oddball task as implemented in our mobile app WearCAAT. All desired features were elicited using an abbreviated visual oddball task on a mobile platform, which demonstrated the validity of WearCAAT functionality and synchrony for functional neuroimaging studies.

While future work entails the validation of more tasks implemented in the current iteration of WearCAAT, and comparisons of fNIRS and EEG features for young versus older adults, this work supports the use of mobile platforms for cognitive neuroimaging.

WearCAAT will soon be easily accessible through both Google Play and Apple App Stores. It is our hope that the wide range of reconfigurable neurocognitive tasks, usability, and ease of use with extant neuroimaging setups will enable nontechnical users to leverage mobile pocket laboratories in future studies and begin to answer outstanding questions in ecological validity. We believe the validation of technical ability as reported in this experiment lends confidence to the pocket lab paradigm and informs future studies into human behavior, in and out in the wild.

## Abbreviations

BRANY: Biomedical Research Alliance of New York
Cz: central midline
EEG: electroencephalogram
ERP: event-related potential
fNIRS: functional near-infrared spectroscopy
HbO: oxygenated hemoglobin
LSL: lab streaming layer
PFC: prefrontal cortex
Pz: parietal midline
ROI: region of interest
RT: response time
WearCAAT: Wearable Cognitive Assessment and Augmentation Toolkit
